# Aberrant expression of long non-coding RNAs and their regulatory role in chromatin-mediated gene expression changes in the prefrontal cortex of major depressive disorder subjects

**DOI:** 10.1038/s41380-025-03396-0

**Published:** 2025-12-18

**Authors:** Yogesh Dwivedi, Bhaskar Roy

**Affiliations:** Department of Psychiatry and Behavioral Neurobiology, Heersink School of Medicine, University of Alabama at Birmingham, Birmingham, AL 35294 USA

**Keywords:** Neuroscience, Depression

## Abstract

Long non-coding RNAs (lncRNAs) have emerged as critical regulators of gene expression, particularly in complex neuropsychiatric disorders such as major depressive disorder (MDD). This study investigates the expression of lncRNAs in the dorsolateral prefrontal cortex (dlPFC) of MDD subjects and their potential roles in chromatin remodeling and gene silencing. Following the 8×60 K microarray platform, we profiled the expression of 35,003 lncRNAs in 59 MDD and 41 control subjects, identifying 1625 upregulated and 1439 downregulated lncRNAs in the MDD group. Co-expression network analysis revealed a complex and interconnected lncRNA network in MDD, suggesting intricate regulatory mechanisms. Furthermore, by employing the PIRCh-seq technique, we found that a subset of 60 upregulated lncRNAs in the MDD brain interacts with heterochromatic regions marked by the H3K27me3 modification, thereby silencing gene expression. These lncRNAs were associated with 24 downregulated protein-coding genes linked to neuronal functions, including synaptic vesicle exocytosis and neurotransmitter release. Gene ontology and pathway analyses highlighted disruptions in critical neurobiological functions, with particular emphasis on synaptic and neuronal signaling pathways. Our findings underscore the role of lncRNA-mediated heterochromatization in the pathophysiology of MDD, offering novel insights into the epigenetic regulation of brain function and behavior.

## Introduction

Major depressive disorder (MDD) is one of the leading causes of global disability, characterized by a poor quality of life, significant disability, and morbidity in affected individuals [1–4]. Despite the availability of a broad array of antidepressants, remission rates among MDD patients remain very low [5, 6]. Furthermore, MDD frequently increases an individual’s risk of suicidal thoughts and behaviors as well as non-suicidal self-injury [7]. Therefore, there is an urgent need to identify the biological substrates of MDD to develop effective treatments.

It is now well accepted that MDD involves both short- and long-term maladaptive processes in response to external stimuli, which impair individuals’ ability to interact appropriately with their environment [8, 9]. The fine-tuning of gene regulation through gene-environment interactions is central to both adaptive and maladaptive processes. In this regard, research over the past decade has provided strong support for the importance of epigenetic mechanisms in the pathogenesis of MDD [10–17]. Recently, long non-coding RNAs (lncRNAs), a burgeoning class of molecules broadly defined as RNA transcripts <200 nucleotides with no protein-coding potential, have emerged as significant epigenetic modifiers capable of regulating over 70% of genes in humans [18, 19]. lncRNAs are highly sensitive to environmental cues and are well integrated into complex, environmentally mediated gene expression programs [20]. Their discovery has provided a paradigm shift in understanding the fine-tuning of cellular processes, particularly in regulating neighboring protein-coding genes that play a pivotal role in disease development progression [17, 21].

Although the role of lncRNAs has been established in various brain functions, including neurogenesis, brain patterning, and synaptic and neural plasticity [19, 22–24], their contribution to psychiatric illnesses has only recently begun to be explored. In rodent brains, we were the first to report the differential regulation of lncRNAs in the brains of rats showing depression-like behavior [25, 26]. Very recently, in these rats, we integrated lncRNA and messenger RNAs (mRNAs) and identified molecules specifically associated with resiliency and susceptibility to depression and antidepressant response [27]. In a similar line of investigation, a distinct set of lncRNAs has been noted in the hippocampus of depression-susceptible, anxiety-susceptible, and insusceptible rat subpopulations [28]. One study showed that the lower expression of lncRNA *TCONS 00019174* in the hippocampus was linked to depression-like behaviors in mice [29]. In a postmortem brain study, nine lncRNAs were found to be differentially expressed in the anterior cingulate cortex of MDD subjects; of those, RP1-269M15.3, was affected by a depression-associated SNP [30]. Few studies in peripheral blood also suggest that lncRNAs may serve as biomarkers for MDD [31, 32]. Interestingly, a genetic study showed that rs12526133 and rs2272260 SNPs present in *LINC01108* and *LINC00998*, respectively, could be responsible for their expression levels in opposite directions and may be linked to MDD pathogenesis [33].

The growing significance of lncRNAs in shaping chromatin architecture underscores their role in regulating gene expression through chromatin remodeling and looping. Since most lncRNAs reside in the nucleus, they possess a unique ability to interact with chromatin and recruit chromatin modifiers to the promoters of their target genes, thereby activating or inhibiting gene expression [34, 35]. For instance, lincRNA HOTTIP, which is transcribed from the 5’ tip of the HOXA locus, plays a critical role in coordinating gene activation [36]. HOTTIP facilitates chromosomal looping to bring target genes into close proximity, enabling interaction with the chromatin modifier WDR5/MLL complex, which promotes histone modifications and enhances gene transcription. This ability of lincRNAs to mediate chromatin modifications and long-range gene activation emphasizes their importance in chromatin organization and offers critical insights into their roles in development and disease. Given that the fine-tuning of transcriptional regulation by gene-environment interactions is central to the etiology of MDD and that lncRNAs may contribute to higher-order brain functions through epigenetic reprogramming, it is crucial to examine not only their expression patterns in the MDD brain but also their interactions with chromatin in regulating gene expression. Investigating these interactions in the context of MDD may uncover unique regulatory pathways and potential therapeutic targets. Here, we hypothesize that in the brain of MDD subjects, stress-induced upregulation of lncRNAs may mediate chromatin modifications, leading to heterochromatin formation and downregulation of target genes involved in mood regulation.

In this study, we examined the genome-wide expression levels of lncRNAs in the dorsolateral prefrontal cortex (dlPFC) of MDD and nonpsychiatric control subjects, a critical brain area implicated in mood regulation, decision-making, and emotion control [37]. Additionally, we utilized pull-down assays for interacting RNAs combined with chromatin immunoprecipitation followed by sequencing (PIRCh-seq) to investigate the lncRNAs that interact with specific chromatin marks. We also assessed the functional changes influenced by altered chromatin-associated lncRNAs, specifically those whose expression was silenced. The results of our study provide evidence not only of aberrant regulation of lncRNA but also, for the first time, of their impact on chromatin-associated gene expression and mediated functional changes in MDD brain.

## Materials & methods

### Human postmortem brain samples

Samples from dlPFC (Brodmann area [BA] 9) were obtained from the Maryland Brain Collection at the Maryland Psychiatric Research Center, Baltimore, MD. The cohort comprised 100 individuals, including 59 diagnosed with MDD and 41 non-psychiatric controls (hereafter referred to as controls). Our MDD cohort was further stratified into non-suicide (n = 32) and suicide cases (n = 27). All tissues from controls and MDD subjects were screened for evidence of neuropathology and were excluded if they exhibited features of Alzheimer’s disease, infarctions, demyelinating diseases, or atrophy (or clinical history of these disorders). Toxicology and the presence of antidepressants were examined by analysis of urine and blood samples from these subjects. Brain pH was measured as described previously [38]. The psychiatric diagnosis was determined by Psychological autopsy as described earlier [39] using Diagnostic Evaluation After Death (DEAD) [40] and the Structured Clinical Interview for the DSM-V (SCID) [41]. The demographic and clinical characteristics of the study cohorts are presented in Table S1. The cohort included 41 control subjects and 59 MDD subjects. The two groups were well matched for age (controls: 49.46 ± 2.84 years; MDD: 47.71 ± 2.26 years; p = 0.63), postmortem interval (PMI) (controls: 18.26 ± 0.92 hr; MDD: 20.66 ± 2.07 hr; p = 0.36), RNA integrity number (RIN) (controls: 7.68 ± 0.04; MDD: 7.71 ± 0.03; p = 0.54), and brain pH (controls: 7.10 ± 0.03; MDD: 7.07 ± 0.02; p = 0.40). The gender distribution was comparable between groups (controls: 26 males, 15 females; MDD: 35 males, 24 females). Similarly, there were no significant group differences in race distribution (controls: 5 black, 36 whites; MDD: 6 black, 1 Asian, and 52 whites) or cause of death categories. None of the subjects had documented neurological or neuropathological disorders, ensuring that findings were not confounded by unrelated brain pathology. As expected, antidepressant toxicology at the time of death was observed exclusively in the MDD group, with 31 subjects (52%) testing positive, compared with none in controls. In addition, 4 of 59 MDD subjects (6.77%) showed evidence of substance abuse (3 subjects with alcohol abuse and 1 with cocaine abuse), while no control subjects met this criterion. Taken together, these results indicate that the control and MDD cohorts were well matched across key demographic and tissue quality variables, with group differences primarily reflecting clinical features such as antidepressant exposure and substance use. Detailed psychological autopsy procedures, toxicology assessments, neuropathology, and brain dissection are provided in the Supplementary section.

### RNA isolation

Total RNA was isolated following TRIzol (Invitrogen) method as described earlier [42] and detailed in the Supplemental section. RNA quality and integrity were assessed using an Agilent Bioanalyzer 2100, with samples exhibiting an RIN ≥ 7 selected for downstream analyses.

### LncRNA expression microarray

In this study, we used the Arraystar Human LncRNA Microarray v5.0, a proprietary platform exclusively designed and developed by Arraystar Inc. (Rockville, USA) to provide comprehensive profiling of lncRNAs together with protein-coding genes. This new-generation array platform enables the detection of 39,317 human lncRNAs, including 8393 highly curated Gold Standard lncRNAs and 30,924 Reliable lncRNAs, along with 21,174 protein-coding transcripts, thereby offering unmatched transcriptome coverage. Its design is based on Arraystar’s proprietary lncRNA transcriptome databases, which systematically integrate major public repositories such as FANTOM5 CAT, GENCODE, RefSeq, BIGTranscriptome, knownGene, LncRNAdb, LncRNAWiki, RNAdb, NRED, CLS FL, NONCODE, and MiTranscriptome, complemented by Arraystar’s own discovery pipeline built from over 47 terabases of RNA-seq data and continuous knowledge-based mining of the literature. Each transcript is represented by carefully designed exon- or splice junction-specific probes to ensure isoform-level resolution, high specificity, and detection accuracy, while built-in positive controls (housekeeping genes) and negative controls safeguard the quality and reproducibility of hybridization.

### Microarray hybridization

Total RNA (500 ng) from each sample was labeled using the Arraystar Flash RNA Labeling Kit (Arraystar Inc., Rockville, USA) and hybridized to the expression array slides following the manufacturer’s instructions. After hybridization, slides were washed to remove any non-specifically bound cRNA and were scanned using an Agilent G2505C Microarray Scanner. The raw intensity data were extracted using Agilent Feature Extraction software (version 11.0.1.1).

### Data preprocessing, cleansing, and normalization

The raw microarray data were preprocessed and normalized using the GeneSpring GX v12.1 software (Agilent Technologies, Santa Clara, USA). Background correction was applied, followed by quantile normalization to standardize expression values across all samples. Probes with low signal intensities, defined as those below the 20^th^ percentile, were filtered out. Expression data for each sample were log2-transformed to ensure a consistent distribution of intensity values across all samples, thereby facilitating uniformity in downstream analysis.

### Differential expression analysis

Following normalization, the differential expression of lncRNAs between the control and MDD groups was determined using a t-test. LncRNAs with an absolute fold change >2 were selected for further analysis. Statistical significance was determined using the p-value obtained from the t-test, with a threshold of p < 0.05. To control for multiple comparisons, the False Discovery Rate (FDR) was adjusted using the Benjamini-Hochberg method [43]. LncRNAs with a p-value < 0.05 were considered significantly differentially expressed and selected for further biological interpretation.

### Localization of lncRNAs on chromosomes with phenogram

Using PhenoGram (http://visualization.ritchielab.org/phenograms/plot), the top significantly upregulated lncRNAs were mapped across 22 autosomes. Additionally, the Manhattan plot (https://jee-hyoung-kim-9.shinyapps.io/Manhattan_Plot/) was used to visualize the chromosome-wide distribution of lncRNAs.

### Tissue-specific expression of Key lncRNAs based on genotype-tissue expression database (GTEx)

Brain-specific expression profiles of key upregulated lncRNAs were examined using the GTEx database, confirming their specific expression in the cortical region of the brain and, more specifically, in the frontal cortex to highlight their relevance to brain function. Notably, we leveraged the GTEx resource to independently validate tissue specificity for candidates emerging from our dataset. Procedurally, we queried the GTEx expression profiles for the 60 lncRNAs prioritized in our analysis and assessed their relative enrichment across tissues.

### Covariate analyses

Age, PMI, brain pH, and RIN were correlated with the top 25 differentially regulated lncRNAs in the MDD groups using the Pearson correlation coefficient. The effect of sex, race, antidepressant toxicology, and suicide was also evaluated by comparing the control group with the MDD group.

### PIRCh-sequencing-Based lncRNA enrichment analysis on close chromatin domain

To investigate lncRNAs interacting with specific chromatin marks, PIRCh-seq (Pull-down of Interacting RNAs with Chromatin Immunoprecipitation followed by sequencing) was employed. Initially, chromatin was isolated from dlPFC using a protocol described earlier [44]. The quality of the chromatin was verified by DNA quantification and agarose gel electrophoresis. Next, chromatin immunoprecipitation (ChIP) was performed as described previously [45] using an antibody specific to the histone modification H3K27me3, a marker of facultative heterochromatin, to immunoprecipitate chromatin regions enriched for this modification. Following ChIP, RNA was extracted from the chromatin complexes to capture the lncRNAs interacting with the H3K27me3-marked chromatin regions. The quality of the extracted RNA was assessed using spectrophotometry and agarose gel electrophoresis. RNA sequencing was performed on an Illumina platform to generate paired-end reads for downstream analysis.

The raw sequencing data were processed to align the reads to the human genome (GRCh38). Differential expression was conducted using DESeq2 to identify lncRNAs significantly associated with the H3K27me3 mark in MDD subjects compared to controls, applying a fold-change threshold (log2FC > 1.5) and p-value < 0.05 for statistical significance. This criterion was chosen to focus on differences that are both statistically significant and biologically meaningful, ensuring that the reported candidates reflect substantial expression changes unlikely to arise from minor variations.

### Co-expression analysis

Co-expression relationships among the differentially expressed lncRNAs were evaluated using Pearson correlation coefficients. Significant correlations (R > 0.7, p = 0.01) were used to construct a co-expression network, visualized using Cytoscape. The clustering of lncRNAs into functional groups was performed based on their correlation patterns using hierarchical clustering with average linkage.

### Microarray assay for coding gene: sample preparation, hybridization, and labeling

The microarray assay for coding genes (mRNA) was performed using the same RNA samples used for the lncRNA microarray. Sample preparation and microarray hybridization followed the manufacturer’s standard protocols with minor modifications. Total RNA from each sample was amplified and transcribed into fluorescently labeled complementary RNA (cRNA) using the Arraystar Flash RNA Labeling Kit (Arraystar, Rockville, USA). The random priming method was employed to ensure uniform labeling across the entire transcript, minimizing potential 3’ bias. The labeled cRNAs were hybridized onto the same array chip we have used for the lncRNA expression array. The microchip was embedded with both lncRNA and mRNA-specific probes (Human MicroArray v5.0; 8 x 60 K, Arraystar) for labeled cRNA hybridization. After hybridization, slides were washed thoroughly to remove non-specifically bound cRNA, and the arrays were scanned using the Agilent Scanner G2505C.

### Data analysis: normalization, differential expression, and statistical evaluation

Agilent Feature Extraction software (version 11.0.1.1) was used to analyze the acquired array images. Raw intensity values were subjected to quantile normalization using the GeneSpring GX v12.1 (Agilent Technologies) to ensure uniform data distribution. Normalized expression levels were log2-transformed for subsequent analysis. Differentially expressed mRNAs between the two groups were identified using a Fold-Change filtering method, where the absolute fold change was calculated as the ratio of expression levels between the groups. Statistical significance was determined using a t-test, and the FDR was estimated using the Benjamini-Hochberg method [43] to control for multiple comparisons. Genes with significant differences in expression, as indicated by p-values, were identified for further analysis.

### Expression correlation analysis

Pearson and Spearman correlation analyses were performed to assess the direction and strength of expression correlations between lncRNA and mRNA expression data. Correlation coefficients (R) and statistical significance (p) values were calculated for each lncRNA-mRNA pair. A correlation was considered noteworthy if it met the threshold of |R|≥0.3 and approached statistical significance at p < 0.05.

### Functional enrichment analysis

Gene Ontology (GO) analysis was conducted using DAVID (http://david.abcc.ncifcrf.gov/) to assess the functional role of the coding genes that have been screened as described in the following section. For this analysis, only genes near H3K27me3-silenced chromatin domains, which are associated with 60 lncRNAs, were considered. This approach allowed for the evaluation of their role in biological processes, their contribution to cellular components, and their influence on molecular functions relevant to MDD-associated pathophysiology. GO terms with p < 0.05 were considered statistically significant. In the GO analysis, we followed standard procedures, including the selection of biological processes (BP), molecular functions (MF), and cellular components (CC) associated with the genes. A Fisher’s Exact test was applied to evaluate the significance of term enrichment, and FDR correction was used to control for multiple testing. Additionally, pathway analysis was performed using KEGG to map the identified genes to relevant signaling pathways. Further, to map the pathways in a connected network, we used the GeneMANIA online tool available in the public domain. KEGG pathway analysis typically involves determining the enrichment of pathways based on the distribution of genes in known biological functions, with significance determined by a threshold of p < 0.05, using similar statistical tests as in the GO analysis.

## Results

### Effects of confounding variables on the expression of lncRNAs in the MDD group

As shown in Figure S1, PMI, brain pH, and RIN values were not significantly correlated with the top 25 significantly altered lncRNAs in the MDD group, except for four lncRNAs in relation to age. Similarly, antidepressant toxicology, suicide, and substance abuse had no significant effects on these 25 lncRNAs (Figures S2-S4).

### Profiling LncRNA expression in the dlPFC of MDD and control subjects

**Fig. 1:**
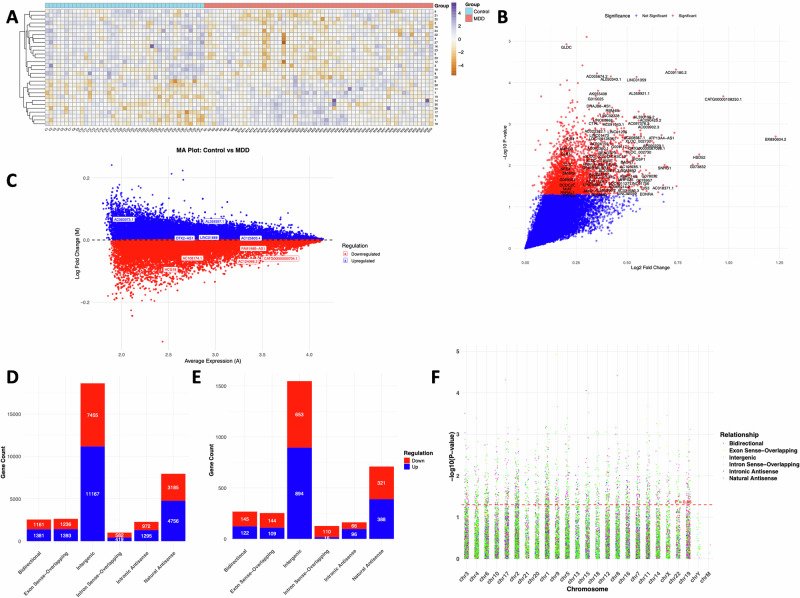
Profiling of lncRNA following the microarray platform and their biotyping in the dlPFC of non-psychiatric control and MDD subjects. **A** An expression heatmap showing normalized expression values of lncRNAs was determined across the sample based on a group-wise comparison between non-psychiatric controls (n = 41) and MDD subjects (n = 59). A cluster dendrogram has been added by clustering the 30 differentially expressed lncRNAs across the samples based on their hierarchical clustering following similarities in their expression pattern. **B** and **C** Volcano and MA-plots from microarray expression data analysis. Red dots indicate significantly upregulated lncRNAs from microarray data analysis results. **D** The stacked bar diagram displays the upregulated and downregulated lncRNAs, grouped by their respective biotypes. The biotyping included bidirectional, axon-sense overlapping, intergenic, intron-sense overlapping, intronic antisense, and natural antisense categories in MDD. **E** The stacked bar diagram displays the significantly upregulated and downregulated lncRNAs, grouped by their respective biotypes. The biotyping included bidirectional, axon-sense overlapping, intergenic, intron-sense overlapping, intronic antisense, and natural antisense categories in MDD. **F** Manhattan plot showing the expression log2 fold change (log2FC) of differentially expressed lncRNAs across various biotype classifications, illustrating the relationship between biotype and expression level in MDD.

To identify the differential expression of lncRNAs in MDD, we profiled their expression patterns in the dlPFC of 59 subjects from the MDD group and 41 subjects from the control group. We found that 1625 lncRNAs were significantly upregulated and 1439 lncRNAs were significantly downregulated in the MDD group compared to the control group (p < 0.05) (Tables S2 and S3). A heatmap of the top 30 differentially expressed lncRNAs is shown in Fig. 1A, which demonstrates distinct expression clusters that separate the MDD group from the control group. A volcano plot (Fig. 1B) illustrates the distribution of 35,003 differentially expressed lncRNAs. The red colored dots show a total of 3064 significantly differentially regulated lncRNAs following p < 0.05. Some of the top significantly altered lncRNAs in the MDD group are labeled in the volcano plot. The differential expression changes based on mean normalized counts are presented with an MA plot (Fig. 1C), where the differences were transformed into a log2 fold-change (log2FC) scale. The MA plot reveals patterns of upregulation and downregulation, with the most significant lncRNAs showing higher expression in the MDD group.

**Fig. 2:**
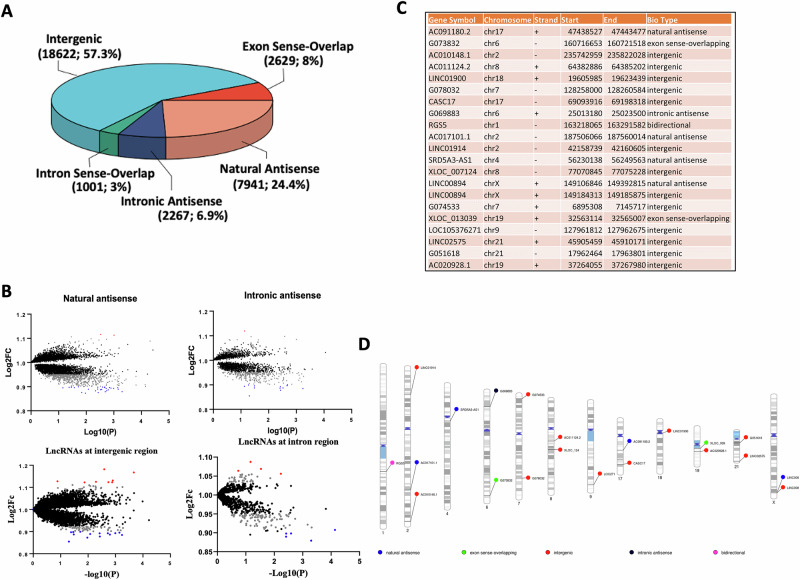
MDD-specific lncRNA biotyping based on transcript distribution and karyotype. **A** Pie chart illustrates the transcriptome-wide distribution of five distinct classes of long non-coding RNAs, identified through expression microarray data in the MDD brain. Each segment of the pie plot represents the total number of lncRNAs identified in their respective classes. **B** MA plots based on expression values of lncRNAs in MDD subjects demonstrating their respective transcriptional origin in the genome. The expression data identified a large proportion of expressed lncRNAs, which were assigned under two broad categories, i.e., natural antisense and intronic antisense. However, based on the expression data, they were also largely classified into three different categories, including exonic, intergenic, and intronic regions. **C** Top 21 differentially expressed lncRNAs with ≥1.2-fold expression upregulation in MDD brain. The table also demonstrates the biotype of individual lncRNAs, which was found to be significantly upregulated in the MDD group compared to the control group. **D** Phenogram displaying the chromosome-wise mapping of the top 21 significantly upregulated lncRNAs in MDD. The lncRNAs are labeled on individual chromosomes, with color annotations indicating their respective biotypes.

Understanding the different lncRNA biotypes is crucial for interpreting their distinct functional and regulatory roles. When characterized by genomic location, orientation, and relationship to protein-coding genes, lncRNAs exhibited a wide variety of biotypes. A stacked bar diagram shown in Fig. 1D illustrates all the differentially (upregulated and downregulated) regulated lncRNAs categorized by their biotype. We have also presented Fig. 1E to display the significantly differentially regulated lncRNAs and their biotype. These included bidirectional, axon-sense overlapping, intergenic, intron-sense overlapping, intronic antisense, and natural antisense. Additionally, a Manhattan plot was generated to visualize log2FC of differentially expressed lncRNAs with the various biotype classifications (Fig. 1F), illustrating the relationship between the biotype and expression level of lncRNAs. Further, the lncRNA transcript proportion for each biotype is summarized in Fig. 2A by showing transcript counts and also their % proportion. As shown, most lncRNAs belonged to intergenic (18622; 57.3%), followed by natural antisense (7941; 24.4%), exon sense overlapping (2629; 8%), and intronic antisense (2267; 6.9%). Only a fraction of lncRNAs belonged to intron sense overlapping (1001; 3%). Further, each class of key biotypes highlighted for their regulatory role in the epigenomic domain is presented as an individual MA plot with normalized expression in the log2FC scale (Fig. 2B).

In this study, we primarily focused on lncRNAs that were significantly upregulated in the PFC of MDD subjects. These lncRNAs can interact with silenced heterochromatic regions marked by trimethylation of histone H3 at lysine 27 (H3K27me3). The top 21 MDD-associated significantly upregulated lncRNAs (>1.5-fold), their chromosomal origin, and transcriptional coordinates, including the start and end sites, are presented in Fig. 2C. The table also highlights the biotype classification of each lncRNA based on its genomic position, orientation, and proximity to nearby protein-coding genes. We also mapped the relative positions of the top 21 significantly upregulated lncRNAs across 22 autosomes and sex chromosomes (Fig. 2D). As shown in the phenogram, the lncRNAs were distributed across 11 autosomes and the X chromosome. The majority of the lncRNAs were located on the long (q) arm of the chromosomes. Generally, these lncRNAs were telomeric, except for lncRNAs SRD5A3-AS, LINC01900, XLOC_039, and G051618 on chromosomes 4, 18, 19, and 21, respectively.

### Co-expression network analysis of lncRNAs in the dlPFC of MDD and control subjects

**Fig. 3:**
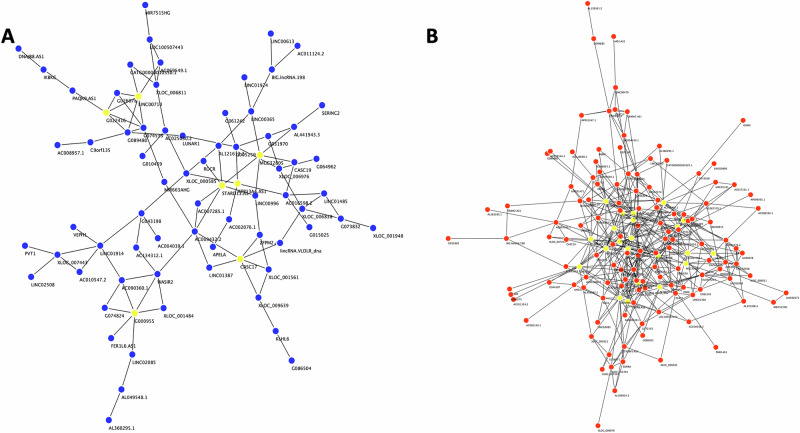
Differential co-expression network of lncRNAs displayed independently in control and MDD groups. Connectivity networks based on lncRNA co-expression analysis show topological differences in inter nodal connectivity between control **(A)** and MDD **(B)** specific effects of differential lncRNA expression. lncRNAs were mapped in the two respective networks based on their degree of connections with other lncRNAs within the network. The respective hub lncRNAs from the two networks are highlighted in yellow.

Co-expression analyses were conducted to explore potential interactions among significantly upregulated 257 lncRNAs in the MDD and control groups separately (threshold: R > 0.7 and p < 0.01) (Fig. 3A and B). Significant co-expression relationships were identified following the correlation threshold, as mentioned in the method section. In the MDD group, 478 lncRNA-lncRNA interactions formed a complex network, whereas only 106 interactions were observed in the control group. The networks exhibited prominent modules with clusters based on correlation patterns. The topological features of the co-expression networks reveal differences between the MDD and control groups regarding the strength and number of lncRNAs involved. The MDD group exhibited a more complex network with a greater number of interactions, as shown by the denser network and more intersecting edges. In contrast, the control group had fewer lncRNAs, reflecting a simpler interaction network. These findings imply that the MDD group displays a more intricate lncRNA interaction network, potentially resulting in complex regulatory control over downstream target genes. The co-expression networks also demonstrated a scale-free topology, featuring a small number of lncRNAs serving as highly connected hubs. In the control group, G022416, MGC32805, LINC00710, ATP13A4-AS1, CASC17 G00095, and STARD were the most highly connected and co-expressed lncRNAs. Conversely, the MDD group contained several highly connected hub lncRNAs, including MGC32805, AC025030.2, XLOC_011375, AL022318.1, LOC105376271, DNAJB8.AS1, AC134312.1, AC007285.1, LUNAR1, G034853, LYRM4.AS1, AC018467.1, XLOC_001948, LINC00334, G040996, G043256, STARD13.AS, G049958, G042650, LINC01638, AC092941.1, XLOC_007124.

### Heterochromatin-associated lncRNA signatures in MDD revealed by PIRCh-seq analysis

**Fig. 4:**
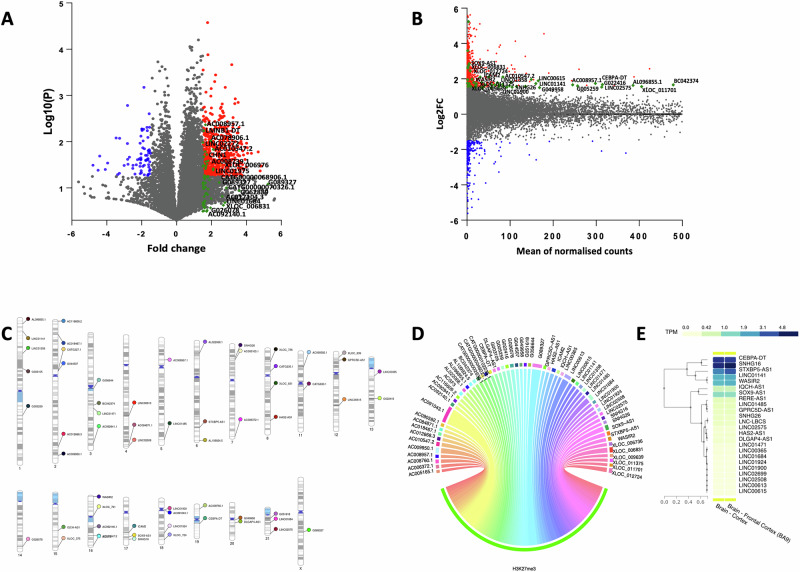
PIRCh-sequencing data analysis following fold change calculation based on normalized read counts. **A,** **B** Both panels represent the normalized read count-based differential expression profile of lncRNAs as determined in the MDD group compared to the control group. **A** The right-side panel is the Volcano plot for PIRCH-seq data analysis, representing relative enrichment and depletion of lncRNAs in the immuno pull-down (H3K27-antibody) complex on both sides of the central plot axis. Red dots on the right side of the plot are significantly upregulated lncRNAs, whereas green dots highlight 60 lncRNAs that were found enriched or upregulated in both PIRCH-seq results and lncRNA microarray data. **B** The left side panel is the MA plot with a similar representation of lncRNA expression data, but with a log-transformed fold change on the Y-axis of the plot. Randomly selected lncRNAs from the list of 60 are labelled in both plots. **C** Phenogram displaying the chromosome-wise mapping of the top 60 significantly upregulated lncRNAs in MDD across 19 autosomes and one allosome (Chr X). The lncRNAs are labeled on individual chromosomes, with color annotations indicating the names of the respective lncRNAs. **D** Top 20 lncRNAs associated with PIRCH-seq data showing their enriched interaction with H3K27me3-modified histone protein are presented with a chord diagram. **E** Human brain tissue enriched expression profile of 24 matched lncRNAs as found in Genotype-Tissue Expression (GTEx) database.

PIRCh-seq is a powerful technique that enables the identification of lncRNAs interacting with specific chromatin marks. In this study, we utilized this technique to investigate the role of lncRNAs in the repressive chromatin environment, with particular emphasis on the H3K27me3 modification, a hallmark of chromatin silencing regulated by the PRC2-EZH2 complex. By using PIRCh-seq enrichment scores, we mapped 3908 lncRNAs (with a FC ≥ 1.5) that were linked to the repressive histone modification. Figure 4A and B present the differential enrichment and depletion of lncRNAs between control and MDD groups, visualized through a volcano plot illustrating fold change values and an MA plot mapping log2FC. The red dots on the volcano plot highlight the lncRNAs significantly upregulated in the MDD group. From PIRCh-seq data, we identified 60 lncRNAs significantly associated with the H3K27me3 mark in the MDD group. The 60 lncRNAs also matched the list of significantly upregulated lncRNAs from our microarray expression data. A list of these 60 lncRNAs and their characteristics is shown in Table S4. In Fig. 4C, we mapped the 60 lncRNAs across 19 autosomes and one sex chromosome (Chr X). From the phenogram, it is evident that the lncRNAs are sparsely distributed among the autosomes and allosomes. However, chromosomes 1, 2, and 16 harbored a maximum number of lncRNAs on both arms (p & q). Additionally, a chord diagram in Fig. 4D highlights 60 individual lncRNAs and their interactions with the H3K27me3 histone mark on the repressed chromatin. Based on these findings, we show that these 60 lncRNAs interact with chromatin in a combinatorial pattern, predominantly enriching heterochromatin regions marked by H3K27me3. This enrichment indicates their potential role in recruiting the PRC2-EZH2 complex, a crucial epigenetic regulator, to silence gene transcription by targeting specific genomic loci and trimethylating histone H3 lysine residue 27. Furthermore, we validated the brain-focused expression enrichment of 60 lncRNAs using the human GTEx expression database. Among these, 24 lncRNAs displayed notable cortical enrichment, specifically in the prefrontal cortical area (Fig. 4E and Table S5).

### Profiling and expression analysis of mRNA transcripts to determine functional changes influenced by altered lncRNAs via heterochromatinization

**Fig. 5:**
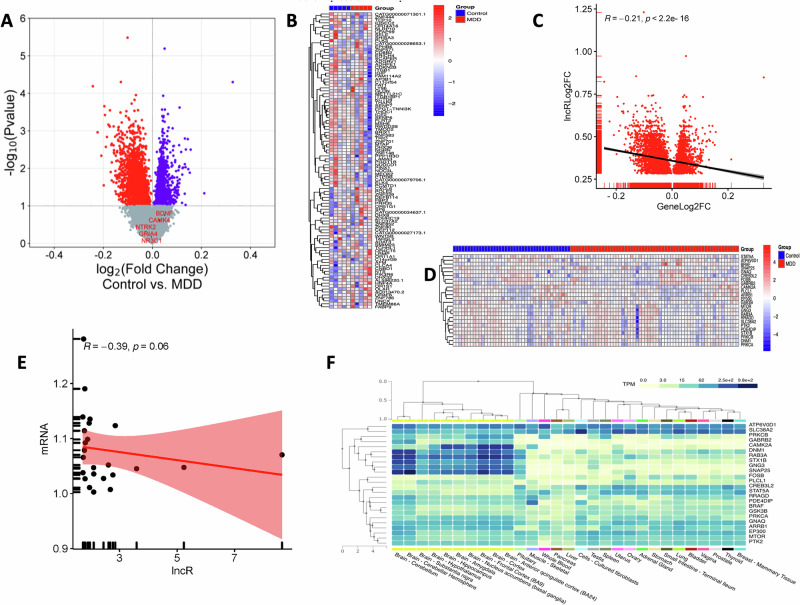
MDD-specific expression profiling of coding genes following the microarray platform. **A** Volcano plot showing differential expression profile of all up- and downregulated coding genes as determined in the dlPFC of MDD and control subjects. Upregulated genes are presented with blue dots on the right side of the plot axis; on the left axis, the downregulated genes are shown with red dots. **B** Expression heatmap showing normalized expression values of top 100 coding genes (up and down-regulated) determined across the samples based on group-wise comparison between control and MDD groups (shown for n = 5/group to maintain the clarity of the plot). **C** Scatter plot representing Pearson correlation between lncRNA differential expression (lncRLog2FC) and mRNA differential expression (GeneLog2FC) in the MDD group. Scatter plot and trend line (Pearson’s correlation) showing a statistically significant inverse correlation between lncRNA expression and gene expression changes, as presented with log2 fold change values. The line represents a linear regression. **D** Expression heatmap showing normalized expression values of 24 downregulated coding genes found in the vicinity of silenced chromatin based on group-wise comparison between healthy control and MDD groups. **E** Scatter plot representing Spearman correlation between 60 lncRNA differential expression (lncR) and 24 mRNA differential expression (mRNA) in MDD group. Scatter plot and trend line (Spearman correlation) showing an inverse correlation between lncRNA expression and gene expression changes as presented with log2 fold change values. The line represents a linear regression. **F** Brain-Specific Expression Enrichment of 24 downregulated genes in the context of other tissues was examined using the GTEx database. GTEx aggregates data from tissue-specific gene expression across a spectrum of non-diseased tissue sites from over 1000 individuals.

To understand the relationship between lncRNA-mediated heterochromatization and gene expression, we investigated how these molecular changes influence the functional roles of coding genes in the MDD brain. Expression microarray was performed on the dlPFC samples from MDD and control subjects used for lncRNA array and PIRCh-seq analyses (Tables S6 and S7). The differential expression profile of up- and downregulated genes is presented with a Volcano plot in Fig. 5A. It shows 1279 downregulated and 733 upregulated genes in the MDD group. A list of these significantly altered genes is provided in Table S8. Furthermore, a heatmap of the top 100 upregulated and downregulated coding genes (Fig. 5B) was generated to visualize the distinct expression patterns in MDD compared to the control group. Based on Pearson correlation analysis, we have also found a significantly inverse (R = −0.21, p < 0.005) relationship between transcriptome-wide gene expression changes and the differential lncRNA expression in the MDD group (Fig. 5C). Next, we identified 24 downregulated genes, shown as an expression heatmap in Fig. 5D, from the list of 2012 significantly differentially regulated genes in the MDD group that were located near silenced heterochromatin domains, which were previously linked to 60 upregulated heterochromatin-enriched lncRNAs in the PIRCh-seq data. We also determined concordance between the low transcriptional output of these 24 coding genes and chromatin condensation. Following Spearman’s method, an inverse correlation (R = −0.39) between the 60 lncRNAs and the 24 coding genes was observed in the MDD group, which was very close to significance (p = 0.06) (Fig. 5E). We would like to emphasize that following Spearman’s correlation analysis, a moderate negative monotonic trend was detected between the 60 lncRNAs and 24 coding genes in the MDD group. However, this association did not reach statistical significance, and the inverse pattern is subtle in the scatter plot (Fig. 5E). Additionally, the inverse trend observed between the upregulated lncRNA and nearby downregulated mRNAs in Fig. 5E should be interpreted as a biologically constrained relationship rather than a predictive model. The regulatory influence of lncRNAs is likely limited by the local chromatin environment, as these mRNAs are positioned near silenced heterochromatic domains, and by compensatory network-level mechanisms that buffer gene expression. These factors may explain why the effect did not extend in a strictly linear fashion but instead reflects a context-dependent ceiling on regulatory impact. Furthermore, our tissue-specific expression analysis using the GTEx database confirmed brain-specific enrichment of these 24 key genes involved in lncRNA-mediated heterochromatization, highlighting their potential roles in regulating brain function and behavior (Fig. 5F).

### Gene function enrichment analysis

**Fig. 6:**
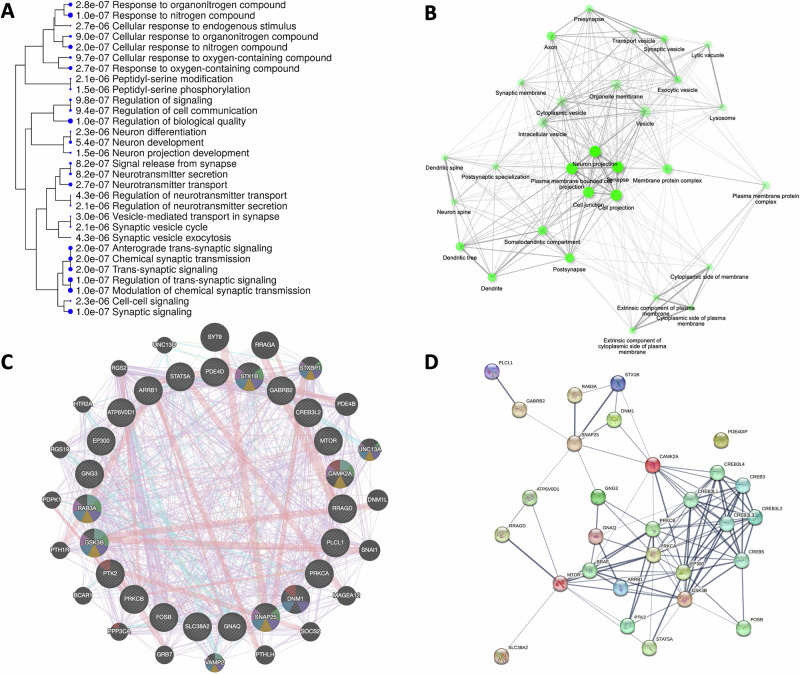
Functional clustering and analysis of 24 downregulated genes in MDD based on their close physical association with transcriptionally silenced chromatin domains across the genome. **A** Gene ontology (GO) enrichment plot showing the significantly impacted ontological functions associated with 24 downregulated coding genes mapped in the vicinity of closed chromatin domains across the genome. **B** Pathway-oriented connectivity network based on 24 closed chromatin-associated mRNA coding genes. The network feature extracted the maximum degree of connectivity for the functions related to neuron projection and synaptic ability. The network clearly highlighted the role of pre- and post-synaptic membranes based on the 24 repressed chromatin-associated downregulated genes in the MDD brain. **C** Connectivity network showing the relationships between 24 input genes. This is a composite of all of the networks chosen from the GeneMANIA database in a way that best connects related genes. In this composite network, the nodes represent genes, and links or edges represent internetwork connectivity. **D** PPi (protein-protein interaction) network based on the direct (physical) and indirect (functional) associations of 24 downregulated genes as obtained from the STRING database.

To further investigate the functional significance of 24 chromatin-associated downregulated genes, we performed computational analysis, including gene ontology and pathway predictions. we found potential disruptions in several critical neurobiological functions, such as synaptic vesicle cycle, synaptic vesicle exocytosis, synaptic and trans-synaptic signaling, and extracellular vesicle formation, as well as neuron development, differentiation, projection, neurotransmitter release, and transport (Fig. 6A). In pathway analysis, we developed connectivity network-focused pathways, which revealed the higher degree of connectivity for functions associated with neuronal spine architecture, neuron projections, dendritic spine formation, somatodendritic compartment configuration, and a variety of pre- and postsynaptic functions. Importantly, the network emphasized the critical role of pre- and postsynaptic membranes regarding the downregulated genes linked with closed chromatin regions in the MDD group (Fig. 6B). We further investigated the relationships among the 24 downregulated genes and other gene family members with similar functionality by constructing a composite network from the GeneMANIA database, which effectively connects genes based on shared functionalities (Fig. 6C). In this composite network, nodes represent genes, and edges indicate the connectivity between genes. The underlying algorithm weighted the network based on genetic interactions, pathways, co-expression, co-localization, and protein domain similarity, providing a measure of how informative the network is for the specific set of input genes. The algorithm highlighted several key genes that strongly drove network functionality, including GSK3B, CAMK2A, SNAP25, RAB3A, STX1B, STXBP1, UNC13A, and VAMP2. These genes were found to have the highest association with the network’s connectivity and were central to the observed biological conditions.

To further refine the strength of gene-gene interactions, we applied a betweenness filter that removes edges with low betweenness centrality—an indicator of how frequently a gene can serve as a bridge in the network and created a protein-protein interaction network (PPi) plot. With this filter, we could highlight key regulatory interactions, thereby improving the clarity and biological relevance of this network (Fig. 6D). Based on this betweenness centrality analysis, we identified that the most strongly connected genes were CAMK2A, CREB, PRKCB, GSK3B, mTOR, and BRAF.

### Ethics approval and consent to participate

All methods were performed in accordance with the relevant guidelines and regulations. The study was approved by the Institutional Review Board of the University of Alabama at Birmingham (Approval #N130823007). Human brain tissue was collected only after a family member provided written informed consent. Also, permission was obtained from the family member for clinical records to be obtained from mental health treatment providers when there was a prior history of mental health treatment.

## Discussion

This study thoroughly investigated lncRNAs and their influence on chromatin-regulated gene expression changes, as well as their possible roles in transcriptional silencing in the dlPFC of individuals with MDD. This is based on a growing body of evidence indicating that lncRNAs act as epigenetic regulators, modulating chromatin accessibility and gene transcription through their interactions with chromatin-modifying complexes [46]. Our integrative approach, which combines lncRNA microarray, PIRCh-seq, mRNA microarray and bioinformatics analyses, offers novel insights into the functional relevance of lncRNAs and their role in mediating heterochromatinization in the pathophysiology of MDD.

Our lncRNA expression profiling revealed 1625 upregulated and 1439 downregulated lncRNAs in the dlPFC of MDD subjects, demonstrating a large-scale alteration in lncRNA expression in MDD. These changes were not associated with age, sex, PMI, or brain pH. In our findings, it is notable to see a large proportion of the lncRNAs to be upregulated in the MDD brain. This is in contrast to an earlier publication showing a higher percentage of lncRNAs to be downregulated in the rostral cingulate cortex [30]. However, this observed discrepancy can be explained based on several factors. First, regional specificity is likely to play a role: the rostral cingulate cortex and dlPFC subserve distinct functions in mood regulation and cognitive control, and differences in local circuitry, cellular composition, and chromatin states could drive region-dependent transcriptional patterns. Second, the inclusion of suicide as a clinical phenotype in the rostral cingulate cortex cohort may reflect additional layers of molecular pathology distinct from non-suicidal MDD, potentially leading to more pervasive transcriptional downregulation. Third, other cohort-specific variables—including sample size, comorbidities, and methodological differences—may also influence the observed directionality of lncRNA changes. Furthermore, the differences between our findings and those reported by Zhou et al [30]. may be attributed to methodological variations, particularly probe localization and transcript selection. The specific region of the gene targeted by the probe and the particular transcript variants measured can markedly influence expression patterns and, in some cases, lead to opposite results. In addition, the other study used an RNA sequencing strategy. Overall, RNA-seq uses a different analytical approach and sensitivity profile compared to probe-based methods. These methodological differences might therefore account for the contrasting results observed between the two studies. Moreover, we anticipate a significant role of stress axis modulation in the upregulation of lncRNAs in MDD, which may partly reflect intricate molecular mechanisms, particularly the dysregulation of the hypothalamic–pituitary–adrenal (HPA) axis and glucocorticoid receptor (GR) signaling. As a ligand-activated transcription factor, GR can directly regulate the transcription of lncRNAs in response to elevated glucocorticoids. Persistent alterations in GR activity in MDD may thus drive the selective induction of stress-responsive lncRNAs, providing a mechanistic connection between chronic stress and the transcriptional changes observed in this study. Interestingly, in a mouse study, it has been reported that an lncRNA, FEDORA, was significantly associated with depression in females only, and this lncRNA contributed to sex differences in depression [47]. The same group also reported the sex-specific role of lncRNA LINC000473 in MDD [48]. Although we did not find significant differences between males and females, further studies will be needed if sex differences observed in the previous study are brain-region specific. As mentioned earlier, a recent study showed that 13% of lncRNAs were differentially regulated in the rostral cingulate cortex of depressed suicide individuals, of which 60% were downregulated [30]. In an earlier study, we had examined whether alterations in hippocampal lncRNAs were associated with resiliency or susceptibility to developing depression in a rodent model of learned helplessness (LH) [25]. We noted 19 upregulated and 216 downregulated lncRNAs in non-LH (NLH) compared to control rats. In contrast, the comparison between LH and the control group identified 128 upregulated lncRNAs and 199 downregulated lncRNAs. Interestingly, 12 upregulated lncRNAs were uniquely associated with the NLH phenotype, and 122 upregulated lncRNAs with the LH phenotype. Our present and previous studies not only highlight the role of lncRNAs in the pathophysiology of depression but also indicate that their regulation varies across different brain regions.

The distribution of lncRNAs across genomic regions demonstrated that a majority of lncRNAs were associated with the intergenic region ( ~ 57%), followed by antisense (~32%). This is in agreement with previous reports showing similar distribution patterns of lncRNA in human brains [49]. It has been demonstrated that intergenic lncRNAs are involved in modulating chromatin dynamics [50], whereas antisense lncRNAs influence cis-regulatory transcriptional repression [51]. Differential regulation of different classes of lncRNAs in the dlPFC of MDD subjects suggests that they may be involved in regulating genes in a distinct fashion. Moreover, our co-expression network analysis revealed a denser lncRNA interaction network in the MDD group compared to the control group, where the co-expression was relatively sparse. This suggests that non-coding RNAs play a role in large-scale transcriptional coordination in MDD, regulating mood and behavior.

Understanding the relationship between lncRNAs and chromatin organization represents an emerging area of research. Studies in other contexts have provided important insights into the interplay between chromatin architecture and complex gene regulation processes [52]. Because of their localization in the nucleus, lncRNAs have the distinctive ability to associate with chromatin [34, 35]. There, they can interact with and recruit chromatin modifiers to the promoters of their target genes to activate or inhibit their transcription. They can also act as molecular decoys, sequestering specific chromatin modulators from the promoters of target genes [53], or can directly interact with DNA and generate DNA-RNA hybrid structures [46], influencing chromatin accessibility and remodeling. Interestingly, a recent report suggests that lncRNAs implicated in the heterochromatic regulation of genes were dysregulated in peripheral blood mononuclear cells of individuals with psychosis and exhibit expression patterns associated with clinical diagnosis, symptom severity, and antipsychotic treatment [54]. In our study, we used immunoprecipitation-based PIRCh-seq to identify lncRNAs that were associated with chromatin. In the dlPFC of MDD subjects, we identified 60 lncRNAs associated with H3K27me3, a hallmark of transcriptionally silenced chromatin [55]. While multiple histone modifications are associated with heterochromatinization, they mark distinct forms of chromatin regulation. H3K9me2/3 is primarily associated with constitutive heterochromatin, such as repetitive DNA and pericentromeric regions, where repression is maintained in a stable and cell-type–independent manner. In contrast, H3K27me3 is a well-established marker of facultative heterochromatin, reflecting dynamic and context-dependent gene silencing mediated by the polycomb group (PRC) of proteins. Given our specific focus on stress-induced epigenetic changes in the brain, we considered H3K27me3 to be the most appropriate marker for capturing facultative heterochromatinization relevant to transcriptional activity in MDD. Our interest in this study was to understand how lncRNAs interact with this stress-sensitive, epigenetically regulated form of chromatin, which is distinct from the more stable repression mediated by H3K9me2/3. As H3K27me3 is a polycomb-mediated mark critical for maintaining transcriptional silencing, the presence of these 60 lncRNAs at H3K27me3-marked heterochromatic regions suggests a role in reinforcing epigenetic repression. This association is particularly significant because several of the genes proximal to these regions are involved in synaptic structure and function, pointing to a potential mechanism by which lncRNA–chromatin interactions contribute to altered gene expression and synaptic vulnerability in MDD. These findings support the hypothesis that lncRNAs play a role in gene repression via histone modifications, a phenomenon gaining traction in neuropsychiatric disorders [56, 57]. Our results also align with reports that H3K27me3-enriched lncRNAs recruit the PRC2-EZH2 complex to promote heterochromatin formation and transcriptional silencing in neurons [58]. This is particularly relevant to MDD, where disruptions in chromatin remodeling and transcriptional repression have been implicated in synaptic plasticity deficits [59–61]. Interestingly, our chromosomal mapping showed that chromosomes 1, 2, and 16 harbored the highest number of H3K27me3-enriched lncRNAs, which aligns with previous studies highlighting these chromosomes as hotspots for psychiatric disorder-associated epigenetic modifications [62]. Interestingly, a majority of the chromatin associated lncRNAs had their loci on the long arm of the chromosome, and most of them were telomeric. Our findings of lncRNAs in telomeric and long arm chromosomal regions highlight their role in stress-related neuropsychiatric disorders like depression as these regions regulate chromosomal stability, stress responses, and synaptic plasticity [63–65].

LncRNAs could serve as master regulators of transcriptional repression [60, 66]. In this study, we found a significant inverse correlation between lncRNA expression and transcriptome-wide gene expression changes. This negative regulatory relationship supports observations that lncRNAs can suppress mRNA transcription through chromatin looping, transcriptional interference, and RNA-mediated gene silencing [67]. Further, the identification of a specific set of 24 downregulated coding genes near heterochromatin domains further highlights a potential mechanism by which upregulated lncRNAs contribute to transcriptional repression in MDD. Interestingly, our GTEx analysis showed that these 24 coding genes had brain-enriched expression. Altogether, the observed inverse correlation of coding genes with lncRNAs suggests a biologically meaningful interaction, reinforcing the significance of lncRNAs in silencing gene expression.

Our gene ontology analysis of chromatin-associated genes revealed that they are part of the pathways that regulate neuron differentiation, neuronal development, neurotransmitter release and transport, and those related to trans-synaptic signaling and dendritic spine formation, all of which have been strongly implicated in MDD [68, 69]. Importantly, our protein-protein interaction network identified key regulatory genes such as GSK3B, CREB, CAMK2A, SNAP25, FOSB, and mTOR, which are well-documented for their role in synaptic plasticity and mood regulation [70, 71]. These findings correspond with earlier research showing that synaptic dysfunction is a core feature of MDD, often characterized by changes in neurotransmission [72]. It is worth mentioning that stress-induced epigenetic changes, including H3K27me3 enrichment and lncRNA-mediated silencing, have previously been linked to alterations in neuroplasticity [73]. H3K27me3, a polycomb-mediated histone modification, is well established as a repressive chromatin mark that can stabilize long-term transcriptional silencing. Separately, lncRNAs can recruit chromatin-modifying complexes or interact with heterochromatic domains to reinforce gene repression. Both processes have been independently linked to enduring alterations in neuroplasticity following stress exposure. This may include changes in synaptic function and structural remodeling. We believe that the convergence of these mechanisms provides a plausible framework for how enduring transcriptional dysregulation may arise in MDD. In this connection, it is also important to highlight that many of these lncRNAs are located in regions of altered chromatin accessibility across the genome. This also suggests that lncRNA–chromatin interactions may directly impact the regulation of genes central to synaptic stability, or conversely, contribute to synaptic disruptions when dysregulated. These findings point to lncRNAs and their chromatin context as potential therapeutic entry points, where approaches such as small molecules interfering with lncRNA–protein or lncRNA–chromatin interactions could be applied to restore gene expression and synaptic function in MDD.

Overall, our study demonstrates that numerous lncRNAs are differentially expressed in the dlPFC of individuals with MDD. The upregulation of these lncRNAs is linked to chromatin modifications that can promote heterochromatin formation and gene repression. Such complex molecular changes may disrupt transcriptional programs essential for mood regulation and higher-order brain functions. Our present findings for the first time highlight lncRNAs as key regulators in the molecular pathology of MDD and point to their potential as novel therapeutic targets.

While our study provides important insights into the role of lncRNAs in MDD, some limitations need to be addressed. One notable limitation is the lack of brain region-specific analysis, as biological differences between brain regions may lead to distinct lncRNA expression profiles and chromatin remodeling patterns. Additionally, our study did not account for cell-type-specific changes, as the postmortem brain samples contain a heterogeneous mix of neurons and glial cells, which may contribute differently to the observed lncRNA expression and interactions. These considerations will be essential for further elucidating the complex role of lncRNAs in the molecular underpinnings of MDD.

In conclusion, our study not only provides strong evidence for the differential regulation of a large number of lncRNAs but also demonstrates that lncRNA-mediated chromatin regulation may contribute to gene repression in the dlPFC of MDD subjects. Identifying heterochromatin-associated lncRNAs, their inverse correlation with a specific set of downregulated coding genes, and their functional involvement in a diverse array of synaptic signaling further underscores the epigenetic complexity of MDD. In the future, it is necessary to determine whether lncRNA-targeting interventions can reverse synaptic deficits and restore normal gene expression in MDD. Nevertheless, our foundational studies offer a novel framework for exploring the role of lncRNAs, particularly in their interaction with chromatin and their implications for psychiatric disorders such as MDD. Moreover, the potential of lncRNAs as biomarkers warrants significant attention, given that a few studies have established a correlation between lncRNAs and both the diagnosis of MDD and the therapeutic response to antidepressant treatment [74–76]. Finally, findings from this study imply that lncRNAs are not only markers of transcriptional dysregulation in MDD but also active mediators of chromatin remodeling and gene repression in the dlPFC. This mechanism may represent a general principle by which stress and environmental factors reprogram transcription in the brain. Beyond MDD, such lncRNA-driven regulation could be relevant to other psychiatric and neurodegenerative disorders where altered chromatin states and impaired gene expression are central features.

## Supplementary information

Supplementary section

Table S2

Table S3

Table S4

Table S5

Table S6

Table S7

Table S8

## Data Availability

All data generated or analyzed during this study are included in this published article [and its supplementary information files].
