# Supplementary material for: Aberrant expression of long non-coding RNAs and their regulatory role in chromatin-mediated gene expression changes in the prefrontal cortex of major depressive disorder subjects
Source: Mol Psychiatry. 2025 Dec 18;31(5):2698–710. doi: 10.1038/s41380-025-03396-0 (PMC13099372; doi:10.1038/s41380-025-03396-0)
Supplement: Supplementary file 1 — Supplementary section [file 41380_2025_3396_MOESM1_ESM.docx]

**SUPPLEMENTAL SECTION**

**Aberrant expression of long non-coding RNAs and their regulatory role in chromatin-mediated gene expression changes in the prefrontal cortex of major depressive disorder subjects**

Yogesh Dwivedi, Ph.D., Bhaskar Roy, Ph.D.

Department of Psychiatry and Behavioral Neurobiology

Heersink School of Medicine

University of Alabama at Birmingham, Birmingham, Alabama, USA, 35294

**^*^Corresponding author:**

Yogesh Dwivedi, Ph.D.

Distinguished Professor of Psychiatry

Elesabeth Ridgely Shook Endowed Chair for Psychiatric Research

Director of Translational Research, UAB Mood Disorder Program

Co-Director, UAB Depression and Suicide Center

Department of Psychiatry and Behavioral Neurobiology

Heersink School of Medicine

University of Alabama at Birmingham

SC711 Sparks Center

1720 7^th^ Avenue South

Birmingham, Alabama, USA 35242

Email: [ydwivedi@uab.edu](mailto:ydwivedi@uab.edu)

**Human Postmortem Brain Samples**

All methods were performed in accordance with the relevant guidelines and regulations. The study was approved by the Institutional Review Board of the University of Alabama at Birmingham (Approval #N130823007). Human brain tissue was collected only after a family member provided informed consent. Also, permission was obtained from a family member for clinical records to be obtained from mental health treatment providers when there was a prior history of mental health treatment. Samples from dlPFC were obtained from the Maryland Brain Collection at the Maryland Psychiatric Research Center, Baltimore, MD. The cohort comprised 100 individuals, including 59 diagnosed with MDD and 41 non-psychiatric controls (hereafter referred to as controls). Additionally, our MDD cohort was further stratified into non-suicide (n=32) and suicide cases (n=27). All tissues from controls and MDD subjects were screened for evidence of neuropathology and were excluded if they exhibited features of Alzheimer’s disease, infarctions, demyelinating diseases, or atrophy (or clinical history of these disorders). Toxicology and the presence of antidepressants were examined by analysis of urine and blood samples from these subjects. Brain pH was measured as described previously. Detailed demographic and clinical characteristics of subjects are shown in **Table S1**. The psychiatric diagnosis was determined by psychological autopsy as described earlier (1, 2) using Diagnostic Evaluation After Death (DEAD) (3) and the Structured Clinical Interview for the DSM-V (SCID) (4). The interviews were done by a trained psychiatric social worker. Two psychiatrists independently reviewed the write-up from this interview, as well as the SCID that was completed from it, as part of their diagnostic assessment of the case. Diagnoses were made from the data obtained in this interview, medical records from the case, and records from the Medical Examiner's office. The two diagnoses were compared, and discrepancies were resolved by means of a consensus conference. Control subjects were verified as free from mental illnesses using these consensus diagnostic procedures.

After removal from the cranium, the brains were cut into six major pieces (four cerebral cortical lobes, basal ganglia-diencephalon, and lower brain stem-cerebellum), rapidly frozen on dry ice, and stored at −70°C until dissection. During dissection, the frontal lobes were sliced into 1-mm to 1.5-mm thick coronal sections at a temperature between 0°C and 10°C. To keep the samples frozen, the dissections were performed on a metal plate over a container filled with dry ice. The prefrontal cortical samples were cut out of the coronal sections by a fine microdissecting (Graefe) knife under a stereomicroscope with low magnification. The dorsomedial prefrontal cortex (Brodmann's area 9) was taken just dorsal to the frontopolar area, including the most polar portion of the superior and partly the middle frontal gyrus between the superior and intermediate frontal sulci. In the sections of the dissected cortical area, the gray and white matter were separated. The tissues were chopped into smaller pieces and stored at −80°C until use.

All tissues from control subjects and suicide victims were screened for evidence of neuropathology by experienced neuropathologists at each brain collection program. The tissues were examined histologically. Fixed sections of PFC were screened with hematoxylin and eosin (H&E) staining and an antibody to glial fibrillary acid protein. Alzheimer’s disease, infarcts, demyelinating diseases, or atrophy disqualified subjects from the study. In addition, in each case, screening for the presence of human immunodeficiency virus (HIV) was done in blood samples, and all HIV-positive cases were excluded. Toxicology data were obtained by the analysis of urine and blood samples.

| **Table S1: Demographic and clinical characteristics of subjects** | | |
| --- | --- | --- |
|  | **Non-psychiatric Controls** | **MDD Subjects** |
| Number of subjects | 41 | 59 |
| Age (Year) | 49.46±2.84 | 47.71±2.26  (F=0.09, p=0.63, t=0.48, df=98) |
| PMI (Hours) | 18.26±0.92 | 20.66±2.07  (F=1.54, p=0.36, t=-0.92, df=98) |
| RIN | 7.68±0.04 | 7.71+0.03  (F=0.44, p=0.54, t=0.61, df=98) |
| Brain pH | 7.10+0.03 | 7.07+0.02  (F=2.63, p=0.40, t=0.81, df=98) |
| Gender  Males  Females | 26  15 | 35  24 |
| Cause of Death | Pneumonia, accidental chest injury, atherosclerotic cardiovascular disease, cardiac arrhythmia, multiple vehicle accident, embolism, leukemia, morbid obesity, electrocution, multiple injuries, GSW, respiratory failure, cardiopulmonary arrest, complications due to diabetes, upper GI bleed, cardiopulmonary arrest, colon cancer, renal failure | GSW, jumped from height, hanging, CO intoxication, drug overdose, stab wound, atherosclerotic cardiovascular disease, multiple vehicle accident, ketoacidosis, cardiomegaly, seizure, fatty liver, cardiac arrhythmia, hemopericardium, liver failure, leukemia, pulmonary embolism, lymphoma, lung cancer, acute myocardial infarction |
| Race | 5 Black/36 White | 6 Black/1 Asian/52 White |
| Suicide | N/A | 32 non-suicide/27 suicide |
| Neurological/  Neuropathological disorders | None | None |
| Number of subjects showing positive antidepressant toxicology | None | 31 |
| Number of subjects showing substance use | None | 4 |
| Data are the mean ± SEM; The data were analyzed using independent sample t-test. MDD group was compared with the control group. | | |

**
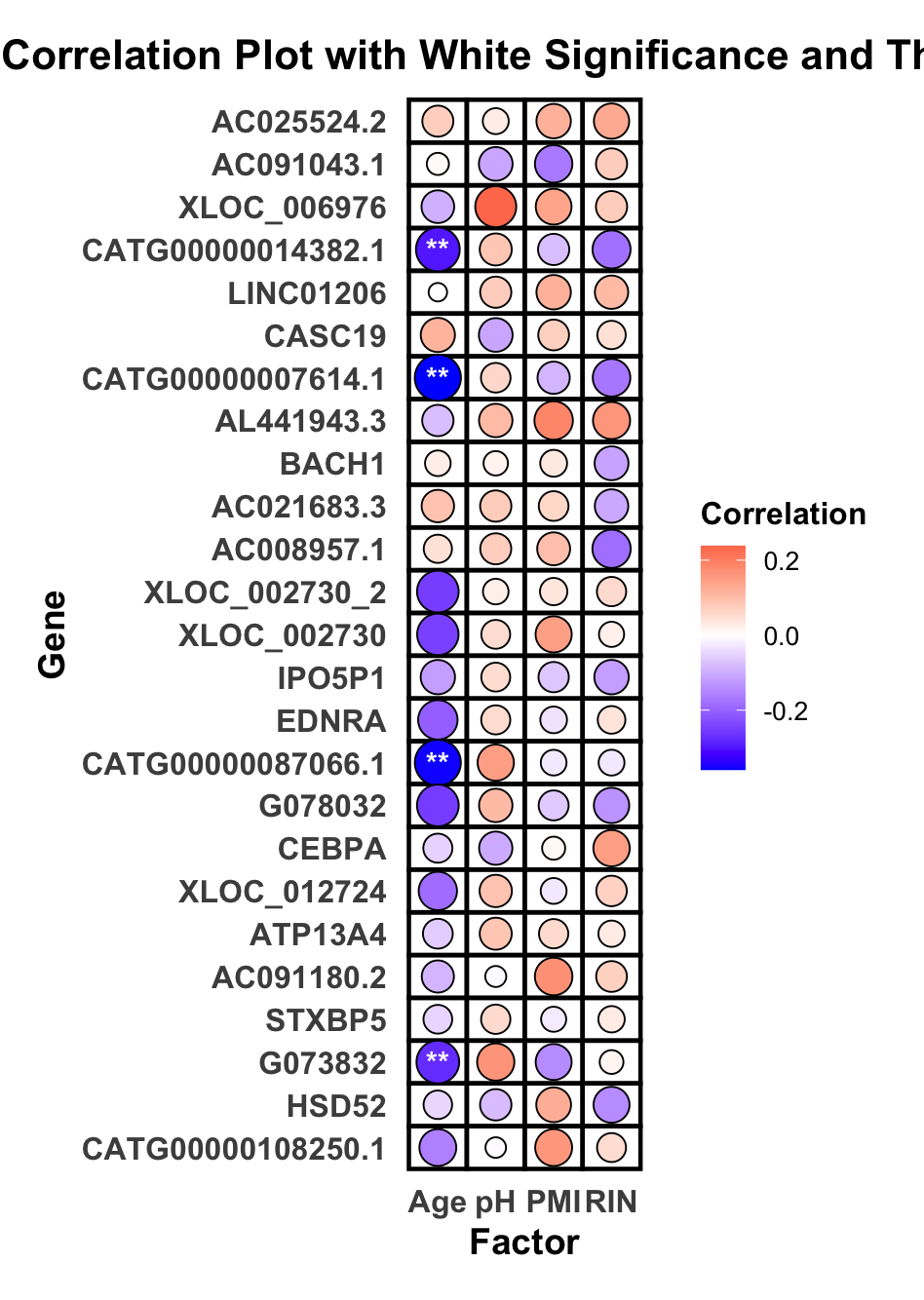
**

**Figure S1:** Effect of age, brain pH, PMI, and RIN on the top 25 significantly upregulated lncRNAs in dlPFC of MDD subjects. The data were analyzed using the Pearson Correlation Coefficient. None of the covariates showed any significant correlation except for four lncRNAs in relation to age (highlighted with a double asterisk symbol).

**Figure S2:** Effect of positive antidepressant toxicology on the top 25 significantly upregulated lncRNAs in dlPFC of MDD subjects. Each bar plot presents data in the mean ± SEM. The level of significance was determined using an independent-sample t-test.

**Figure S3:** Effect of suicide on the top 25 significantly upregulated lncRNAs in dlPFC of MDD subjects. Each bar plot presents data in the mean ± SEM. The level of significance was determined using an independent-sample t-test.

**Figure S4:** Effect of substance use on the top 25 significantly upregulated lncRNAs in dlPFC of MDD subjects. Each bar plot presents data in the mean ± SEM. The level of significance was determined using an independent-sample t-test.

**Reference**

1. Dwivedi Y, Mondal AC, Rizavi HS, Faludi G, Palkovits M, Sarosi A, et al. (2006): Differential and Brain Region–Specific Regulation of Rap-1 and Epac in Depressed Suicide Victims. *Archives of general psychiatry*. 63:639-648.

2. Bhushan D, Yadav J, Rozatkar AR, Moirangthem S, Arora A (2023): The psychological autopsy: An overview of its utility and methodology. *J Neurosci Rural Pract*. 14:447-452.

3. Salzman S, Endicott J, Clayton P, Winokur G (1983): Diagnostic evaluation after death (DEAD). *National Institute of Mental Health, Rockville*.

4. Spitzer RW G, M, First, MD. (1995): *Structural Clinical Interview for DSM-IV (SCID)*. New York, NY: Biometrics Research, New York State Psychiatric Institute.
